# Neonatal Adrenal Hemorrhage Mimicking Suprarenal Tumor in a 12‐Day‐Old With *Escherichia coli* Urosepsis

**DOI:** 10.1155/crpe/2165402

**Published:** 2026-03-01

**Authors:** Khaled Sawafta, Hani Abu Hijleh, Yousef Samara, Tasbeeh Al-Kharraz

**Affiliations:** ^1^ Department of Medicine, An Najah National University, Nablus, State of Palestine, najah.edu

**Keywords:** adrenal cyst, neonatal adrenal hemorrhage, urosepsis

## Abstract

Neonatal adrenal hemorrhage is an uncommon finding that may resemble suprarenal tumors. We describe a term male neonate who developed *Escherichia coli* urosepsis and was incidentally found to have a left suprarenal cystic lesion. Imaging and laboratory workup excluded malignancy, and the infant remained stable under conservative treatment with antibiotics and serial ultrasound follow‐up. This case emphasizes the need to consider adrenal hemorrhage in septic neonates with abdominal masses and supports noninvasive management in the absence of malignant features.

## 1. Introduction

Neonatal adrenal hemorrhage (NAH) is a rare clinical occurrence, with an estimated incidence between 0.2% and 0.55% of live births. It is typically seen in full‐term male infants and is often associated with perinatal stress, sepsis, or hypoxia [1, 2]. Many cases are asymptomatic and detected incidentally, though some may present with an abdominal mass, jaundice, or anemia. Ultrasound is the imaging modality of choice for evaluation [3, 4].

A significant diagnostic challenge is differentiating hemorrhagic adrenal lesions from other suprarenal pathologies, such as adrenal abscess or neonatal neuroblastoma. The imaging similarities between neuroblastoma and NAH—particularly cystic or heterogeneous appearances—necessitate advanced imaging techniques and careful interpretation [5, 6].

This report describes a term male neonate with *E. coli* urosepsis and a left suprarenal cystic lesion, illustrating the diagnostic dilemma between hemorrhagic cyst, abscess, and tumor. The case underscores the importance of a systematic diagnostic approach, multidisciplinary collaboration, and vigilant follow‐up in managing neonatal suprarenal masses.

## 2. Case Presentation

A male neonate was delivered at 38 weeks of gestation via spontaneous vaginal delivery, with a birth weight of 3200 g. The perinatal course was uneventful. At 6 days of age, he presented with fever (38.5°C), poor feeding, and irritability. Laboratory investigations revealed leukocytosis and elevated C‐reactive protein (CRP), consistent with neonatal sepsis. Empirical intravenous antibiotics (ampicillin and cefotaxime) were initiated. Urine culture at 12 days of life yielded *Escherichia coli*, confirming urosepsis.

**FIGURE 1:**
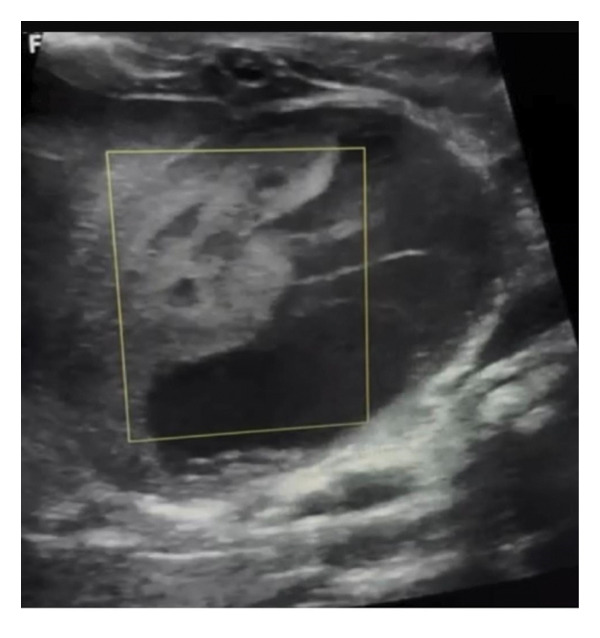
Initial abdominal ultrasound (Day 12) showing a heterogeneous left suprarenal cystic lesion with internal septations, suggestive of adrenal hemorrhage.

**FIGURE 2:**
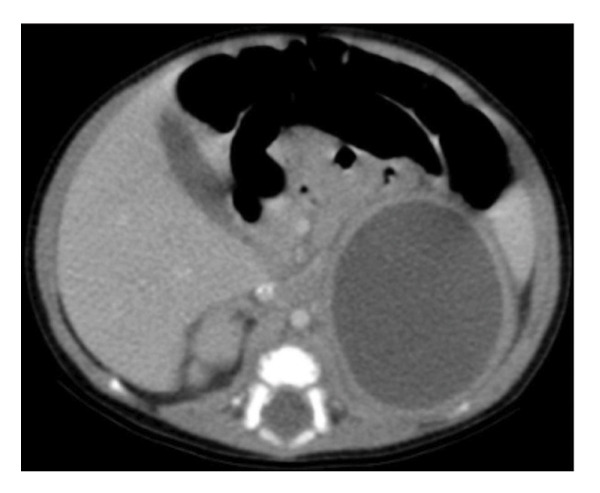
Contrast‐enhanced CT scan (Day 20) showing a left adrenal rim‐enhancing cystic mass without calcification or solid component, consistent with adrenal hemorrhage.

**FIGURE 3:**
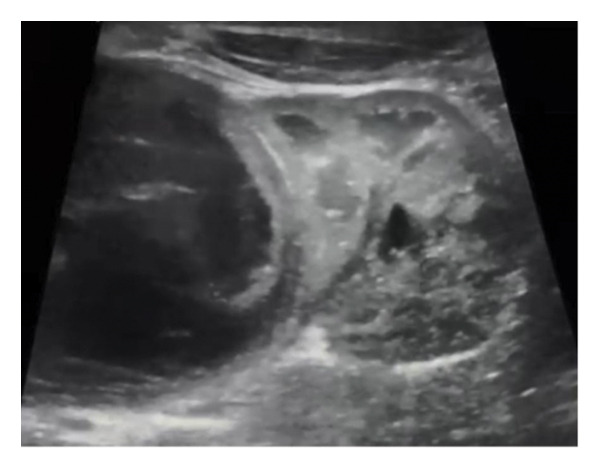
Follow‐up ultrasound (Day 36) showing enlargement and increased heterogeneity of the suprarenal cystic lesion.

**FIGURE 4:**
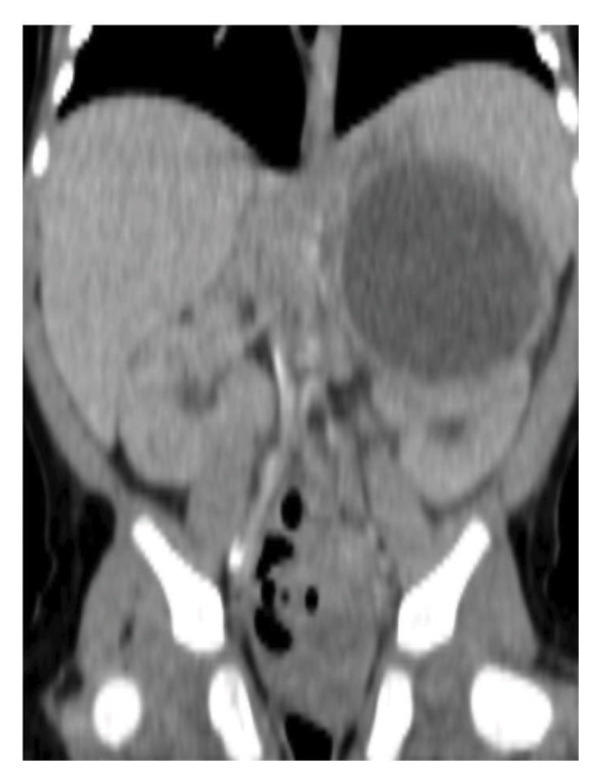
Contrast‐enhanced CT scan (Day 36) showing a cystic suprarenal lesion with heterogeneous internal contents, without enhancement or calcifications, raising suspicion of infected hematoma versus adrenal abscess.

Abdominal ultrasound at presentation revealed a well‐defined, heterogeneous, anechoic left suprarenal lesion measuring 4.0 × 4.5 cm, with internal septations and mild bilateral hydronephrosis (Figure 1), suggestive of adrenal hemorrhage. A contrast‐enhanced CT scan on Day 20 demonstrated a nonenhancing left suprarenal cystic mass with homogeneous low attenuation, without calcifications or vascular invasion (Figure 2), consistent with a hemorrhagic adrenal cyst. Follow‐up ultrasound on Day 36 showed persistence of the lesion with mild enlargement (≈5 × 4 cm) and increased internal heterogeneity (Figure 3). Repeat CT confirmed a cystic suprarenal lesion with heterogeneous contents but no enhancement or calcifications (Figure 4), raising suspicion of infected hematoma versus adrenal abscess.

A second CT scan was performed due to the increase in size and internal heterogeneity observed on ultrasound, aiming to better characterize the lesion and exclude an evolving abscess or necrotic tumor, given the ongoing sepsis.

Serial laboratory follow‐up (Table 1) demonstrated persistent leukocytosis and markedly elevated CRP during the first 3 weeks, with a gradual subsequent decline. The infant developed progressive anemia (hemoglobin decreased from 15.2 to 7.5 g/dL), transient hyperbilirubinemia (peak 11.7 mg/dL), and episodic hypoglycemia (random glucose as low as 50 mg/dL), all of which improved over time. Endocrine evaluation showed normal cortisol and 17‐hydroxyprogesterone levels, with transient elevation of dehydroepiandrosterone (DHEA) that later decreased. Tumor markers (alpha‐fetoprotein [AFP], beta‐human chorionic gonadotropin [β‐hCG], and carbohydrate antigen 19‐9 [CA19‐9]) were within normal limits.

**TABLE 1:** Serial laboratory parameters.

Parameter (reference range)	Day 6	Day 7	Days 9–12	Days 15–20	Days 24–30	Day 36	Day 45
WBC (5.0–21.0 × 10^9^/L)	22.4	20.6	24.6–21.3	19.8–20.4	23.6–13.3	12.5	9.7
CRP (< 5.0 mg/L)	30	+^∗^	44–51	55–91	36–42	21	10
Hgb (13.5–19.5 g/dL)	15.2	−	−	−	↓ to ∼9	8	7.5
Total bilirubin (< 12.0 mg/dL)	11.7	−	10‐11	9‐10	8‐9	7.5	6
Random blood glucose (50–90 mg/dL)	60	50/89	−	−	−	−	89

*Note:* + = positive; − = not tested; Hgb = hemoglobin; WBC = white blood cell count.

Abbreviation: CRP, C‐reactive protein.

^∗^Qualitatively positive.

By 45 days of life, laboratory parameters had nearly normalized. Follow‐up ultrasound at the same age showed a decrease in lesion size and internal heterogeneity, consistent with resolving adrenal hemorrhage. The infant remained clinically stable. A multidisciplinary team including pediatric surgery and infectious disease specialists recommended conservative management with close monitoring, given the absence of malignant features, improving laboratory trends, and overall clinical stability. The clinical timeline is summarized in Table 2.

**TABLE 2:** Timeline of clinical findings, investigations, and results.

Age (days)	Clinical findings	Investigations performed	Results/impressions
6	Fever, suspected sepsis	CBC, CRP, blood culture	Leukocytosis, elevated CRP; blood culture negative
12	Confirmed urosepsis	Urine culture, abdominal ultrasound	*E. coli* isolated; US: left suprarenal cystic lesion (4.0 × 4.5 cm) with mild hydronephrosis
20	Endocrine evaluation	Cortisol, 17‐OHP, DHEA	Cortisol and 17‐OHP normal; DHEA elevated (1800 ng/dL; ref: 50–850 ng/dL)
36	Follow‐up	Repeat US + CT scan	Lesion increased to 5 × 4 cm, heterogeneous; CT: nonenhancing left adrenal cyst, no calcification
40	Tumor marker assessment	AFP, β‐hCG, CA19‐9	All within normal limits
45	Follow‐up	Repeat DHEA	DHEA decreased to 809 ng/dL

*Note:* US = ultrasound; 17‐OHP = 17‐hydroxyprogesterone; DHEA = dehydroepiandrosterone; AFP = alpha‐fetoprotein.

Abbreviations: β‐hCG, beta‐human chorionic gonadotropin; CA19‐9, carbohydrate antigen 19‐9; CT, computed tomography.

## 3. Discussion

This case describes a rare presentation of *E. coli* urosepsis with an associated left adrenal hemorrhagic cyst. NAH occurs more frequently on the right side (≈70% of cases) due to vascular anatomy, with bilateral involvement in about 10% and left‐sided cases in only 10%–15% [7, 8]. Risk factors include birth asphyxia, coagulopathies, traumatic delivery, and systemic infection [7, 8]. In our patient, the adrenal lesion was left‐sided and associated with *E. coli* sepsis without other perinatal insults. Sepsis is a known precipitant of adrenal hemorrhage; though most reported cases are linked to meningococcemia or generalized sepsis rather than urinary sources [8]. A comparison with representative cases is provided in Table 3. Clinical presentation varies: Many cases are asymptomatic, while others manifest as an abdominal mass, jaundice, anemia, or, rarely, adrenal insufficiency [9]. The male predilection observed in this case aligns with previous literature, where NAH is more commonly reported in male neonates, possibly due to hormonal influences or higher birth trauma rates [1, 7].

**TABLE 3:** Comparison with representative neonatal adrenal hemorrhage/abscess cases.

Reference (year)	Age at dx	Context of presentation	Imaging findings	Management	Outcome
Habeb et al. (2014)	4 weeks	UTI workup; incidental adrenal hematoma	Large unilateral adrenal hematoma (left)	Conservative (antibiotics only)	Spontaneous resolution
Rumińska et al. (2015)	3‐4 weeks	Sepsis; presumed adrenal abscess	Complex adrenal cyst/abscess (side NR)	Surgical drainage/exploration	Resolved after surgery
Mandelia et al. (2017)	1‐2 weeks	Perinatal distress; bilateral adrenal abscesses	Bilateral suprarenal cystic masses with debris	US‐guided percutaneous drainage + IV antibiotics	Full recovery
Present case (2025)	6 days	*E. coli* urosepsis; left adrenal hemorrhagic cyst	Left adrenal 4.0 × 4.5 cm cyst with septations, no flow	Antibiotics, watchful waiting	Clinical stability; follow‐up

*Note:* Comparison of neonatal adrenal hemorrhage/abscess cases in the literature (NR = not reported).

The diagnostic approach in this case was thorough, utilizing serial ultrasound, CT, and comprehensive laboratory testing to exclude malignancy and endocrine abnormalities. Although color Doppler ultrasound—a valuable noninvasive tool for distinguishing avascular hematomas from vascularized tumors—was not employed, CT provided detailed anatomical and enhancement characteristics crucial for differentiation in this clinically complex scenario.

Management of neonatal adrenal lesions remains variable. While some cases, particularly abscesses, may require drainage or surgery [10, 11], conservative management with antibiotics and monitoring is appropriate in the absence of liquefied abscess, hemodynamic instability, or malignant features [7, 12]. In our patient, the decision to avoid invasive intervention was supported by clinical improvement, stable imaging findings, and the absence of adrenal insufficiency.

The patient was followed until 45 days of age, by which time significant clinical and radiological improvement was evident. Long‐term follow‐up data are not available due to the family’s relocation; however, the observed trend toward resolution supports a favorable outcome, consistent with the natural history of uncomplicated adrenal hemorrhage.

This case highlights the importance of considering adrenal pathology in septic neonates with abdominal masses and demonstrates the value of a stepwise, multidisciplinary approach. By reporting this case in accordance with SCARE guidelines, we provide a detailed account that may assist clinicians in similar scenarios.

## 4. Conclusion

This case illustrates a rare presentation of NAH associated with *Escherichia coli* urosepsis, initially mimicking a suprarenal tumor. Differentiating benign hemorrhagic lesions from infectious or malignant masses requires a comprehensive, stepwise approach including imaging, endocrine evaluation, tumor markers, and close clinical monitoring. Conservative management with antibiotics and serial imaging successfully averted unnecessary surgical intervention. This report underscores the importance of including adrenal hemorrhage in the differential diagnosis of abdominal masses in septic neonates and supports a noninvasive strategy when malignancy is excluded and the clinical course remains stable [13, 14].

## Author Contributions

Khaled Sawafta collected the clinical data, organized the laboratory and imaging findings, and drafted the initial manuscript.

Hani Abu Hijleh supervised the overall patient management, provided critical input regarding the surgical aspects, and contributed to revising the manuscript.

Yousef Samara assisted in the literature review, interpretation of clinical data, and editing of the manuscript.

Tasbeeh Al‐Kharraz reviewed and interpreted the ultrasound and CT images, prepared the radiological descriptions, and contributed to figure preparation.

## Funding

No specific grant from funding agencies was received for this work.

## Disclosure

All authors have read and approved the final version of the manuscript and agree to be accountable for all aspects of the work.

## Ethics Statement

Our institution does not require ethical approval for reporting individual case reports or case series.

## Consent

Written informed consent was obtained from the patients for their anonymized information to be published in this article.

## Conflicts of Interest

The authors declare no conflicts of interest.

## Data Availability

The data that support the findings of this study are available from the corresponding author upon reasonable request.
